# Mapping the learning styles of medical students in Brazil

**DOI:** 10.1186/s12909-024-05028-7

**Published:** 2024-01-10

**Authors:** Marcel Fernando Inácio Cardozo, Gilmar Cardozo de Jesus, Maria Helena de Sousa, Amilton Iatecola, Fernanda Latorre Melgaço Maia, Gisele Massarani Alexandre de Carvalho, Vinícius Rodrigues Silva, Daniela Vieira Buchaim, Adriane Gonçalves Moura Cardozo, Ronny Rodrigues Correia, Rogerio Leone Buchaim, Marcelo Rodrigues da Cunha

**Affiliations:** 1 Postgraduate Program in Health Sciences, Faculty of Medicine of Jundiaí, 13202-550 Jundiaí, Brazil; 2 Department of Implant Dentistry, Faculdade São Leopoldo Mandic, 13045-755 Campinas, Brazil; 3 Department of Human Anatomy, University of San Francisco (USF), 12916- 900 Bragança Paulista, Brazil; 4 Postgraduate Program in Structural and Functional Interactions in Rehabilitation, University of Marilia (UNIMAR), 17525-902 Marilia, Brazil; 5 Medical School, University Center of Adamantina (UNIFAI), 17800-000 Adamantina, Brazil; 6 Graduate Program in Anatomy of Domestic and Wild Animals, Faculty of Veterinary Medicine and Animal Science (FMVZ), University of Sao Paulo (USP), 05508-270 Sao Paulo, Brazil; 7 Postgraduate Program in Teaching Sciences and Mathematics, University Cruzeiro do Sul, 08060-070 São Paulo, Brazil; 8 Botucatu Medical School (FMB), São Paulo State University (UNESP—Univ Estadual Paulista), 18618-687 Botucatu, Brazil; 9 Department of Biological Sciences, Bauru School of Dentistry, University of Sao Paulo (FOB/USP), 17012-901 Bauru, Brazil; 10 Orthopedics and Traumatology Sector, Faculty of Medicine of Jundiaí, 13202-550 Jundiaí, Brazil

**Keywords:** Teaching, Learning, Learning styles, Teaching methods, Medical education

## Abstract

**Background:**

Medical education has evolved based on the application of pedagogical actions that place the student as the protagonist of the learning process through the use of active teaching methodologies. Within this context, higher education teachers should use strategies that focus on the student and his/her context and avoid traditional teaching methods. Specifically in medical schools, there is an even greater challenge since the teaching methods of medical curricula differ from those used in previous schooling. Consequently, students acquire their own style of processing information that is often incompatible with the profile of medical schools. This may be one of the factors responsible for the lack of motivation among undergraduates.

**Objective:**

The aim of this study was to characterize the learning styles of students enrolled in a Brazilian medical school using the Felder-Soloman Index of Learning Styles (ILS).

**Methods:**

This was a cross-sectional, descriptive, quantitative study that included students from the 1st to the 6th year of a Brazilian medical school. The students participating in this study voluntarily answered 44 questions about learning styles of the Felder-Silverman instrument validated in Brazil. The instrument was divided so that each domain consisted of 11 questions with two response options in which only one could be selected. For each domain, a score (1 point) was assigned to the selected option (a, b) of the question and the learning style category was determined as the difference between these values. For data collection and tabulation, we used the Learning Syle Platform (EdA Platform) developed based on Felder’s studies since this system processes information about the dimension analyzed, the preferred style, and the most striking characteristics of each style.

**Results:**

The results showed that sensing was the preferred learning style of the students, followed by the sequential and visual styles. It was not possible to determine whether gender or age influences the choice of learning methods because of the homogeneity of the results.

**Conclusions:**

The present data will enable teachers of the institution involved in this study to plan pedagogical actions that improve the students’ self-awareness, as well as their teaching-learning skills, by choosing the most adequate active methodologies for the medical education programs considering the individuality of each student and class.

## Introduction

Medical education has evolved based on the application of pedagogical actions that place the student as the protagonist of the learning process through the use of active teaching methodologies [1]. Within this context, higher education teachers should use strategies that focus on the student and his/her context and avoid traditional teaching methods [2, 3].

Active methodologies have enabled reflection on the role of the teacher and the student in the teaching and learning process and seek to promote changes in classrooms that are often based on the traditional teaching model [4]. Specifically in medical schools, there is an even greater challenge since the teaching methods of medical curricula differ from those used in previous schooling [5–7]. Consequently, students acquire their own style of processing information that is often incompatible with the profile of medical schools, which may be one of the factors responsible for the lack of motivation among undergraduates [1].

Motivation and the correct use of active methodologies are essential for academic learning [8] and structuring education according to the student’s learning style is key to developing study skills [9]. It is therefore necessary to use teaching practices that encourage student engagement; however, first the profile of the students must be mapped since the learning process is complex and is not limited to the acquisition of answers or knowledge but rather involves numerous individual and social variables [10].

Studies have reported the influence of individual differences on the learning process [11] and the need to adapt teaching to the profile of each student [12], as well as the difficulty in identifying the student’s individual characteristics in order to establish the best methodological approaches to teaching [13]. Hence, it is clear that individuals use their personal patterns to develop strategies that allow them to achieve their learning goals. Some individuals find it easier to understand theories or models, others will better understand facts and concrete data, and there are those who better assimilate visual information such as figures and diagrams rather than spoken and/or written (verbal) explanations. Some individuals prefer group learning, while others choose to work individually [14].

The factors mentioned above may be directly related to the academic performance of students. Thus, theories and instruments for evaluating the student’s learning style profile have emerged to help select the best teaching-learning methods [11]. Within this context, Felder and Silverman (1988) [15] developed the Index of Learning Styles (ILS) that comprises four dimensions: visual/verbal, sensing/intuitive, active/reflective, and sequential/global. According to these authors, the process of information retention depends on the relationship between and the teaching-learning styles of the student and teacher.

Studies on learning styles aim to understand how students internalize new knowledge considering the affective, cognitive, and physical learning domains [16]. Thus, the learning style can influence academic performance and the development of learning skills [17]; in addition, it provides information so that educators can improve student motivation by developing more efficient educational programs for medical training [18]. Studies in the medical literature suggest that the performance of medical students with different learning styles may be compromised [19]. Therefore, teachers must identify the difficulties of students, as well as failures in the education system, in order to promote necessary learning interventions [20].

The feeling of not knowing how to learn or study is a common problem among medical students and identification of the learning style is therefore necessary since it directly influences the ability to assimilate the enormous amount of information during the medical course [21]. Neglecting the factors mentioned above and other psychological factors in medical curricula can negatively affect not only the learning and study skills of medical students but also the increasing prevalence of depression, distress, and anxiety among these students [22–24].

Designing intervention programs is important to help develop the learning skills of medical students [25]. However, first, we must identify the profile of medical students in order to understand the relationship between distress and competency and its impact on academic performance, dropout rates, and professional development [22].

In an attempt to contribute to the progress of medical education by developing more efficient educational programs according to the individual learning style of each medical student and considering the existing contradictions regarding the relationship between learning styles and academic performance in medical education [1], the aim of this study was to characterize the learning styles of medical students from a medical school in Brazil using the ILS of Felder-Soloman [26].

## Methods

### Study design

A cross-sectional, descriptive, quantitative study was conducted that included medical students enrolled in a medical school in Brazil. The study was approved by the Research Ethics Committee of the Faculty of Medicine of Jundiaí (Ethical Clearance Certificate: 39204820.2.0000.5412) and registered with the National Health Council, Ministry of Health.

### Participants

All medical students (*n* = 708) were invited at the beginning of the 2023 academic year to participate in this study, to collect data on individual learning styles, with the number of students per year of the course being: 1st, *n* = 118; 2nd, *n* = 119; 3rd, *n* = 118; 4th, *n* = 116; 5th, *n* = 120 and 6th, *n* = 117. The response rates for the six years were: 1st: 91%; 2nd: 68%; 3rd: 37%; 4th: 30%; 5th: 25% and 6th: 32%.

### Instrument used and measurement of learning styles

The Felder-Silverman learning style model [16] was employed in this study because it is one of the most used models in the field of medical education [1] and has been proven to be a suitable model for research in medical education [26]. In addition, it was validated by Felder and Spurlin (2005) [27] and its reliability was demonstrated in other studies [18, 26, 28].

The Felder-Silverman ILS is divided into four dimensions with the following two opposing styles (categories): perception (sensing/intuitive, evaluates how the individual responds to the teaching environment), input (visual/verbal, evaluates how information is received), processing (active/reflective, evaluates how information is processed), and understanding (sequential/global, evaluates how the individual understands the information) [16, 26, 29]. In this study were calculated the reliability coefficient for the four dimensions, that were, respectively: 0.511, 0.629, 0.584 and 0.312.

**Fig. 1:**
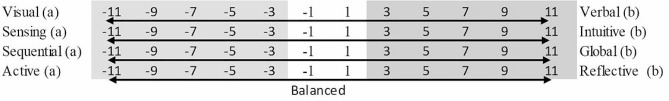
Schematic drawing of the determination of learning styles. The visual, sensing, sequential, and active styles correspond to option “a” of the questions of the Felder instrument according to dimension (domain), while the verbal, intuitive, global, and reflective styles correspond to option “b”. The learning style (category) is determined as the difference between values for each option. The “balanced” category was defined when the value was between − 1 and 1 [30]

The medical students participating in this study voluntarily answered 44 questions regarding the learning styles of the Felder-Silverman instrument validated in Brazil. The instrument was divided so that each domain included 11 questions with two response options in which only one could be selected [15, 16, 30]. Thus, for each domain, a score (1 point) was assigned to the selected option (a, b) of the question and the learning style category was determined as the difference between these values (Fig. 1).

An adaptation described by Jesus (2022) [30] was used for this study. Unlike other studies that used the original instrument to assess learning styles in medical education, this author validated a new category for each domain characterized as “balanced”. Thus, this study considered the four domains with the following styles (categories): visual/balanced/verbal, sensing/balanced/intuitive, sequential/balanced/global, and active/balanced/reflective (Fig. 1).

For data collection and tabulation, the EdA Platform developed by Jesus (2022) [30] based on Felder’s studies was used since this system processes information about the dimension analyzed, the preferred style, and the most striking characteristics of each style.

### Statistical analysis

First, the independent (class, sex, and age) and the dependent variables (four domains), classified into three categories each, were submitted to simple descriptive analysis. Next, bivariate analysis was performed considering each categorized domain according to each independent variable. The following tests were applied: Yates’ chi-square test (with continuity correction) for 2 × 2 tables, Fisher’s exact test (when Yates’ test was not applicable), or Pearson’s chi-square test. The last was used for contingency tables with more than two dimensions. Analysis was performed using the SPSS v.20 software and the level of significance was pre-set at 5%.

To analyze, in statistical tests, the association of factors of differences in learning styles, according to the year of study, the sample n per year was considered. No normality test was used, because the domains/dimensions were analyzed in a categorized manner (in three categories each) and, therefore, a non-parametric statistical test (chi-square test) was used, with a confidence interval (CI) of 95%.

## Results

A total of 335 medical students participated in this study, including 107 (31.9%) from the 1st year, 81 (24.2%) from the 2nd year, 44 (13.1%) from the 3rd year, 35 (10.4%) from the 4th year, 30 (9.0%) from the 5th year, and 38 (11.3%) from the 6th year. There was a higher participation of 1st - and 2nd -year students.

Most participants were female (*n* = 246, 73.4% versus 26.6% males). Regarding the distribution by age group, the mean age of the participants was 22.1 years (SD = 3.97 years), with a minimum age of 18 years and a maximum age of 49 years.

**Fig. 2:**
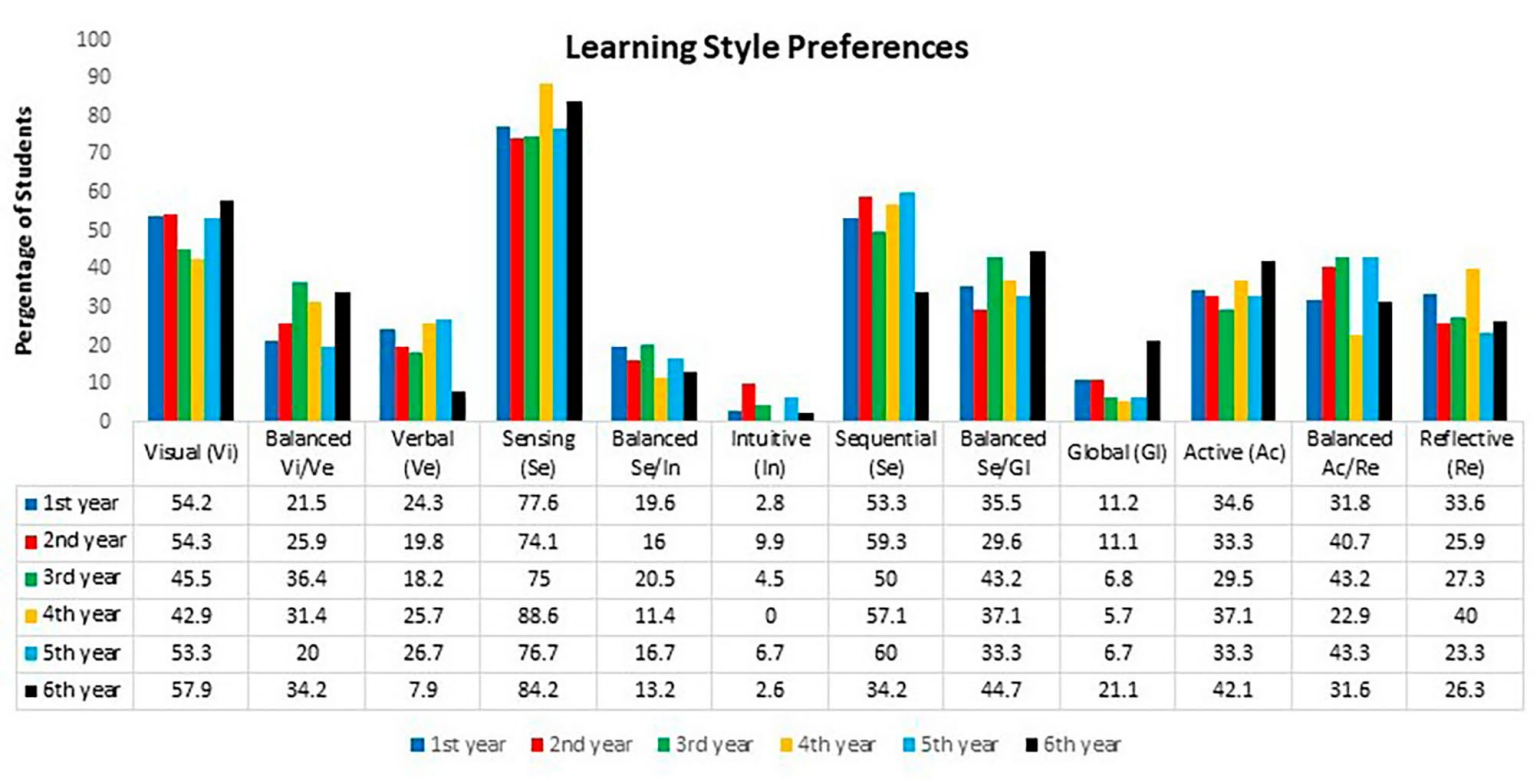
Representation of learning style preferences according to class

**Fig. 3:**
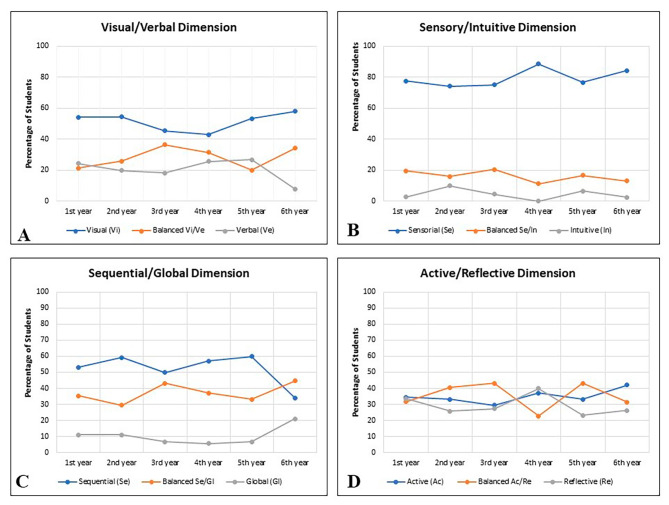
Representation of learning style dimensions

First, the learning styles were analyzed considering the six medical classes separately (1st to 6th year). Regarding the visual/verbal dimension, there was a higher prevalence of the visual style (> 40% in all classes) and no significant difference was observed between classes. Lower percentages were observed for the verbal style. For the balanced category, higher values were found for the 3rd and 6th year, but the visual style predominated (Figs. 2 and 3A). Regarding the sensing/intuitive dimension, there was a predominance of the sensing style (percentages > 74% in all classes), with no significant differences between classes. The percentage of the intuitive style was less than 10% in all classes (Figs. 2 and 3B).

Regarding the sequential/global dimension, there was a predominance of the sequential style from the 1st to the 5th year. The balanced style was the most frequent profile among 6th -year students. Statistical analysis revealed no difference between the 1st - to 5th -year classes but there was a difference compared to the 6th year (*p* = 0.018). The percentage of students with the global style was lower than those of the other two categories in all classes (Figs. 2 and 3C).

Analysis of the active/reflective dimension showed no statistically significant difference in the percentages of the three categories between the 1st - to 6th -year classes. This domain was the most homogeneous in terms of the three response categories (Figs. 2 and 3D).

Table 1 presents the confidence intervals (95% CI) used to estimate learning style preferences by class.

**Table 1:** Data obtained with their respective confidence intervals regarding learning style preferences

	1st year	2nd year	3rd year	4th year	5th year	6th year
Visual (Vi)	54.2 (44.3–63.9)	54.3 (42.9–65.4)	45.5 (30.4–61.2)	42.9 (26.3–60.6)	53.3 (34.3–71.7)	57.9 (40.8–73.7)
Verbal (Ve)	24.3 (16.5–33.5)	19.8 (11.7–30.1)	18.2 (8.2–32.7)	25.7 (12.5–43.3)	26.7 (12.3–45.9)	7.9 (1.7–21.4)
Balanced (Vi/Ve)	21.5 (14.1–30.5)	25.9 (16.8–36.9)	36.4 (22.4–52.2)	31.4 (16.9–49.3)	20 (7.7–38.6)	34.2 (19.6–51.4)
Sensing (Se)	77.6 (68.5–85.1)	74.1 (63.1–83.2)	75 (59.7–86.8)	88.6 (73.3–96.8)	76.7 (57.7–90.1)	84.2 (68.7–94.0)
Intuitive (In)	2.8 (0.6–8.0)	9.9 (4.4–18.5)	4.5 (0.6–15.5)	0 (0.0–10.0)	6.7 (0.8–22.1)	2.6 (0.1–13.8)
Balanced (Se/In)	19.6 (12.6–28.4)	16 (8.8–25.9)	20.5 (9.8–35.3)	11.4 (3.2–26.7)	16.7 (5.6–34.7)	13.2 (4.4–28.1)
Active (Ac)	34.6 (25.6–44.4)	33.3 (23.2–44.7)	29.5 (16.8–45.2)	37.1 (21.5–55.1)	33.3 (17.3–52.8)	42.1 (26.3–59.2)
Reflective (Re)	33.6 (24.8–43.4)	25.9 (16.8–36.9)	27.3 (15.0–42.8)	40 (23.9–57.9)	23.3 (9.9–42.3)	26.3 (13.4–43.1)
Balanced (Ac/Re)	31.8 (23.1–41.5)	40.7 (29.9–52.2)	43.2 (28.3–59.0)	22.9 (10.4–40.1)	43.3 (25.5–62.6)	31.6 (17.5–48.7)
Sequential (Se)	53.3 (43.4–63.0)	59.3 (47.8–70.1)	50 (34.6–65.4)	57.1 (39.4–73.7)	60 (40.6–77.3)	34.2 (19.6–51.4)
Global (Gl)	11.2 (5.9–18.8)	11.1 (5.2–20.0)	6.8 (1.4–18.7)	5.7 (0.7–19.2)	6.7 (0.8–22.1)	21.1 (9.6–37.3)
Balanced (Se/Gl)	35.5 (26.5–45.4)	29.6 (20.0–40.8)	43.2 (28.3–59.0)	37.1 (21.5–55.1)	33.3 (17.3–52.8)	44.7 (28.6–61.7)

**Fig. 4:**
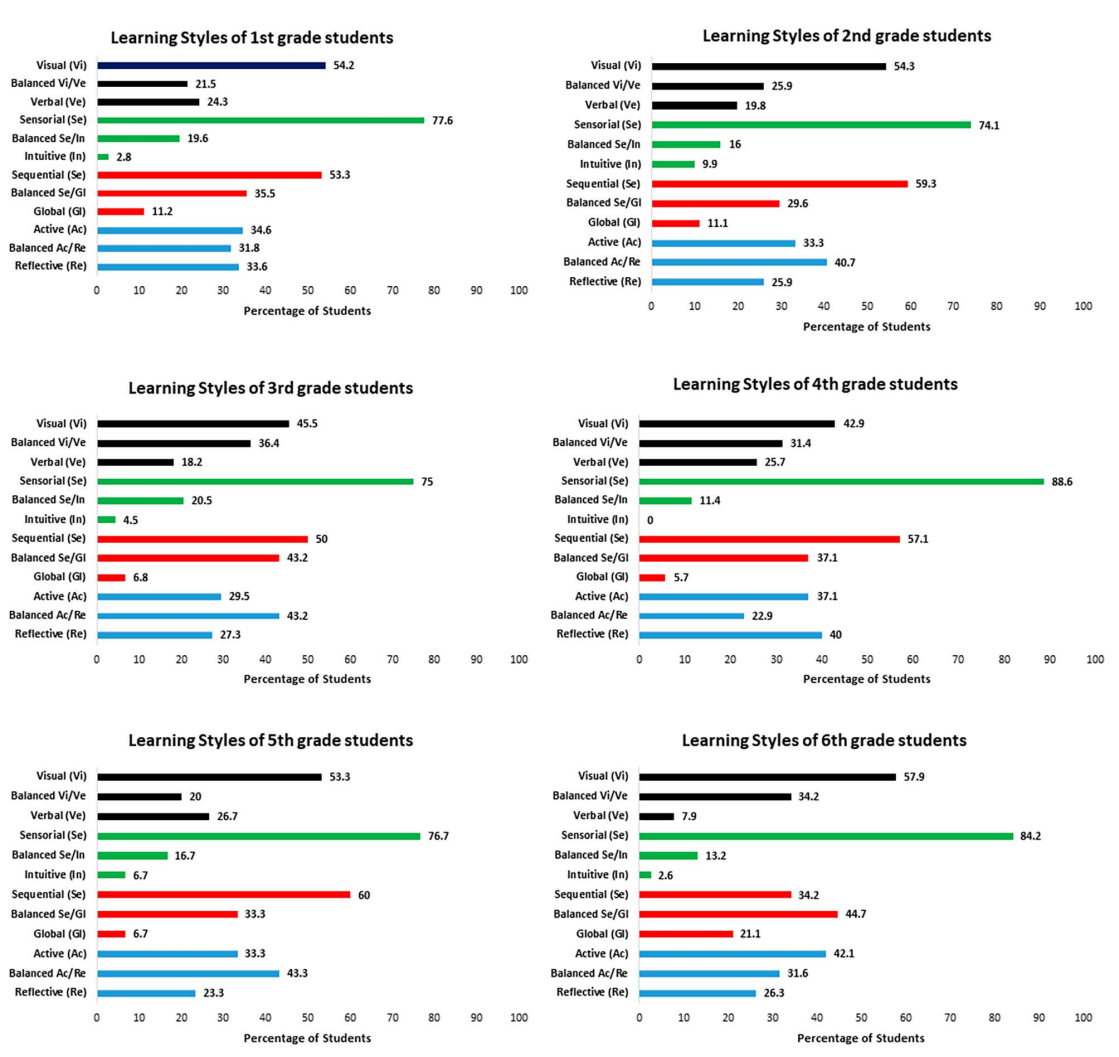
Learning style preferences per class (1^st^ to 6^th^ year)

**Fig. 5:**
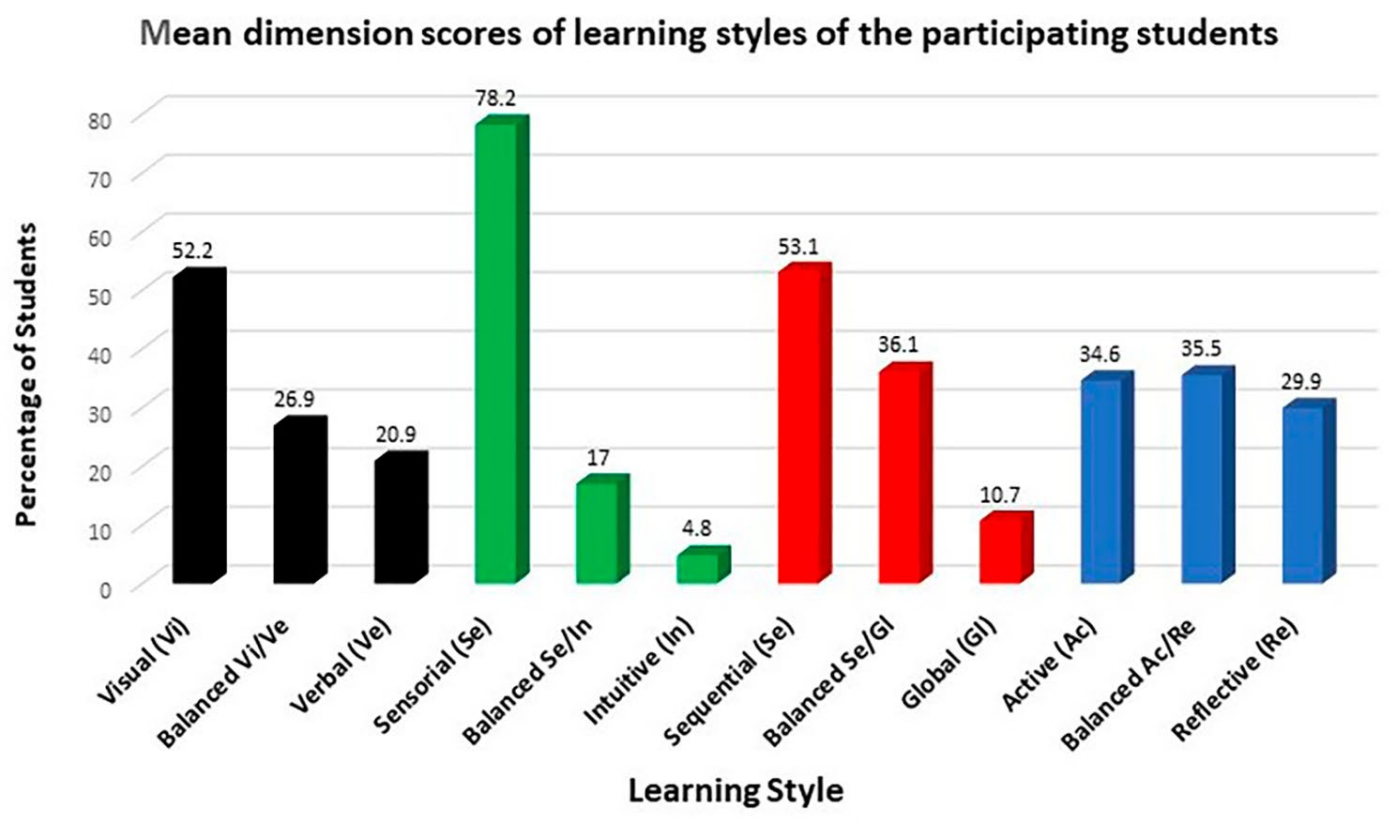
Overall distribution of learning style preferences among medical students

Separate analysis of each dimension according to class and total sample showed that medical students prefer the sensing, visual, and sequential style categories (Figs. 4 and 5). When compared according to the age of the medical students, the percentages of the dimensions studied were similar, except for the sequential style whose percentage was higher among students aged 18 to 21 years and the balanced and global styles whose percentages were lower in this age group (*p* = 0.008) compared to students older than 21 years. Comparison between genders showed a higher percentage of the visual and active styles among male students, while the percentages of the sensing and sequential styles were similar between genders (Table 2).

**Table 2:** Percentual distribution of preferences of learning styles according to participant age and gender

	Age (years)			Gender		
Preference	18–21	≥ 22	*p*-value *	Male	Female	*p*-value *
Domain 1			0.157			**0.032**
Visual (Vi)	48.8	55.8		64.0	48.0	
Balanced (Vi/Ve)	26.2	27.6		21.3	28.9	
Verbal (Ve)	25.0	16.6		14.6	23.2	
Domain 2			0.922			0.861
Sensorial (Se)	77.9	78.5		78.7	78.0	
Balanced (Se/In)	16.9	17.2		15.7	17.5	
Intuitive (In)	5.2	4.3		5.6	4.5	
Domain 3			**0.008**			0.788
Sequential (Se)	61.0	44.8		50.6	54.1	
Balanced (Se/Gl)	31.4	41.1		37.1	35.8	
Global (Gl)	7.6	14.1		12.4	10.2	
Domain 4			0.667			0.098
Active (Ac)	33.1	36.2		43.8	31.3	
Balanced (Ac/Re)	37.8	33.3		29.2	37.8	
Reflective (Re)	29.1	30.7		27.0	30.9	

* Pearson chi-square test

## Discussion

Medical schools are constantly concerned with the teaching-learning process since students are faced with a situation in which they must understand and apply an enormous amount of information within a limited period of time [1]. To solve this problem, it is essential to know the students’ learning styles since this knowledge directly affects the choice of methods used to stimulate their learning skills, which is important for the student’s academic performance; in addition, students will be able to better understand themselves. This may achieve valuable harmony between the teacher and student in the use of the most appropriate pedagogical practices for teaching that are compatible with the individuality of the students’ learning styles [1, 18, 31, 32]. However, there is paucity of specific data on the learning styles of medical students in Brazil and their association with academic performance during medical training.

Numerous studies in medical education have used the Felder-Silverman model to analyze the type of learning style of medical students, which can be characterized as visual or verbal, sensing or intuitive, sequential or global, and active or reflective [1, 18, 26]. Visual learners are students who remember what they see, who like graphs, images and diagrams, and who prefer visual presentations of information. Verbal students remember what they hear or read, like to hear or read information, prefer explanations with words, and learn while listening and talking about the content. Sensing learners use the manipulation of what was explained, are observant and methodical, solve problems using traditional methods, look at the facts, and perceive information in a concrete manner. Intuitive learners use their imagination, are innovative and curious, do not like repetitions, are not detail-oriented, focus on the meaning, and perceive information in an abstract manner [30, 33].

Active learners are students who manipulate objects, perform physical experiments, learn by doing, work in groups, prefer classes that address more practical problems, encourage interaction with the content, and better process information by interacting with the data. Reflective learners refer to students who think about information, evaluate options, learn through analysis, work individually, choose classes that explore the bases of the subject, use content and theories, and prefer to spend time reflecting on the presented information. Sequential learners prefer information that is presented in a linear, ordered and progressive way, like analysis, are interested in detail, and put the details together in order to understand the end result. Finally, the global style refers to students who like a holistic and synthetic approach, who have a better view of the whole, and who first see the big picture and then fill in the details [30, 33].

The impact of gender on learning styles must also be considered [18]. In the present study, male students preferred the visual and active styles, but were balanced between the sensing and sequential styles, when compared to female students. These data agree with some studies that identified men as more visual learners [1, 26, 34]. Other studies reported a predominance of the active style among women [35]. However, Alghasham (2012) [36] and Liu and Liu (2023) [18] did not find significant differences between female and male medical students. Oyeyemi et al. (2019) [34] observed heterogeneity in learning preferences between genders and suggested a combination of appropriate voice and audios with images, photos and visual effects for the teaching process. Thus, since there is no consensus in the literature whether age group or gender interferes with the choice of learning styles, we chose to consider in general the learning styles of the groups analyzed during the medical course.

The most common result of studies on learning style in medical schools is the prioritization of more sensory, visual and sequential information by students [26, 35, 37]. In the study by Liu and Liu (2023) [18], the priorities of the students were perceptive, sequential, and visual. The authors concluded that there is a preference or the sequential and linear use of demonstrations, photos, diagrams, and algorithms during the teaching-learning process [38, 39]. These data agree with the present study that also identified that students from the 1st to the 6th year prefer to receive visual presentations of information instead of resources using explanations with words during the learning process of the medical course. In addition, the students prioritize sensory information over intuitive information, a fact that characterizes them as more observant and detail-oriented individuals who use real facts for the learning process throughout the medical course. The percentages of the intuitive style were lower than those of the balanced style, indicating a low preference for the use of imagination in the perception of information during learning.

Statistical analysis in this study also showed that 6th -year students preferred a balanced style in the sequential/global dimension, in contrast to 1st - to the 5th -year students who mainly preferred the sequential style in the content understanding domain. These data demonstrate that most students prefer to use details of the information to better understand the end result during the learning process and that there is a low preference for the holistic perception of information.

Regarding the reflective/active dimension, the percentages of learning styles was similar between students. In contrast, in the study by Liu and Liu (2023) [18], 1st - to 4th -year medical students process more reflective information instead of active information. Bhalli et al. (2015) [40] assessed the learning style of 77 medical students and concluded that the majority preferred active learning strategies. However, the authors did not find a correlation between learning style preference and the preferred teaching strategies of students.

Contradictory results have been reported in the literature for the reflective/active dimension, with some studies showing that students prefer to process information in a more reflective manner [26, 37], while others prefer to process information more actively [35, 36]. These findings highlight the need to consider an additional category in medical education studies. We therefore included the “balanced” category as described by Jesus (2022) [30] in our study. In contrast, the studies cited above used the original instrument of Felder that only included the two categories for each dimension [1, 18, 26, 35, 37]. This difference is important since it permits a more in-depth reflection on the choice of the most appropriate teaching strategy, especially considering student-centered pedagogical actions.

The implementation of teaching methods with the student as the protagonist is necessary to promote learning, development, and conceptual understanding. These methodologies include those based on constructivist principles, such as Problem-Based Learning (PBL) and Team-Based Learning (TBL) [3]. These methods are widely used in medical courses [41], especially after teaching had to be changed in the face of the COVID-19 pandemic [3, 42, 43]. However, Bastos et al. (2022) [3] also cite the use of the Alternative Methodology of Problem Cases (AMPC) based on PBL, modified by Lopes et al. (2019) [41], and TBL proposed by Michaelsen and Sweet (2008) [44]. AMPC is composed of three stages (preparation, application, and reflection) that contain individual and team attributions based on the resolution of case problems. This methodology differs from PBL and TBL by valuing the knowledge and socio-historical background of individuals. It thus enables the development of creativity, curiosity, autonomy, and critical thinking of students that will prepare them for real clinical situations of their future profession [3].

Van der Veken et al. (2008) [45] evaluated student learning patterns in three types of medical curricula: conventional, integrated contextual, and PBL. The authors observed that the problem-based model promoted less learning and a lower ability to express content when compared to students of the conventional curriculum. Students of the integrated contextual curriculum showed a better ability to integrate different aspects into a whole.

Based on the principles of these active teaching-learning methods and on the data obtained in this study in which medical students were found to prefer the sensing and sequential style, as well as considering a certain heterogeneity in the percentage of the balanced category in the domains evaluated, we believe that the AMPC method might be a viable alternative to be tested in future pedagogical actions in medical schools [46–48]. Furthermore, Bastos et al. (2022) [3] also reported the possibility of adaptations in the AMPC method according to the reality of each higher education institution.

As a limitation of this study, we can consider the unequal distribution of students by year, since medical students in the first years are more willing to take part in research, as they spend more time in college with teachers and researchers, compared to students in the last years, especially the last two years, who are interning in health care units or hospitals.

## Conclusion

The preferred learning style of medical students from a medical school in Brazil was the sensing style, followed by the sequential and visual styles. It was not possible to determine whether gender or age may influence the choice of learning methods because of the homogeneity of the results. The present data will enable teachers of the institution involved in this study to plan pedagogical actions that improve the students’ self-awareness, as well as their teaching-learning skills, by choosing the most adequate active methodologies for the medical education programs considering the individuality of each student and class.

## Data Availability

All data generated or analysed during this study are included in this published article.

## Abbreviations

ILS: Felder-Soloman Index of Learning Styles
EdA: Platform Learning Syle Platform
SPSS: Statistical Package for Social Science for Windows
SD: Standard deviation
AMPC: Alternative Methodology of Problem Cases
PBL: Problem-Based Learning
TBL: Team-Based Learning
